# Coronary Artery Fistula and Pectus Excavatum Paradox

**DOI:** 10.1155/2021/6628900

**Published:** 2021-03-03

**Authors:** Mohammad Hashim Jilani, Hina Khawar Jamali, Fahad Waqar, Mohamed Effat

**Affiliations:** ^1^Department of Medicine, Carle Foundation Hospital, Urbana IL, USA; ^2^Division of Cardiovascular Health and Disease, University of Cincinnati College of Medicine, Cincinnati OH, USA

## Abstract

Pectus excavatum is the most common congenital chest wall deformity. Its effects on cardiopulmonary function, exercise capacity, and body image are variable across affected patients. Management practices for pectus deformity vary considerably, but most authors agree on the need for surgical correction if pectus index is >3.0 and there is evidence of cardiac compression on imaging. We encountered a case of a middle-aged man with severe pectus deformity and a coincidental large coronary artery to right atrium fistula. Despite a pectus index of 4.8 and severe right heart compression on thoracic imaging, he had not developed any symptoms or hemodynamic complication from this pectus deformity. Additionally, hemodynamic studies revealed normal left and right heart function, normal pulmonary artery pressures, and absence of any evidence of myocardial ischemia or significant left-to-right shunt. These abnormalities would have been expected with a coronary fistula of this size. His pectus deformity and coronary fistula had opposing hemodynamic effects, thus protecting him from severe complications of either. Presently, an association between congenital coronary fistulae and pectus excavatum is not known, and this is one of the very first reported cases of these two congenital abnormalities coexisting in a patient. Additionally, concurrence of these two conditions poses a unique therapeutic challenge due to their opposing hemodynamic effects.

## 1. Introduction

Pectus excavatum is a common congenital deformity which, in its severe form, can cause significant cardiac dysfunction via right heart compression. Coronary fistulae are rare congenital malformations, and large size fistulae are associated with significant shunt physiology as well as cardiac ischemia. These two congenital deformities have not been reported to coexist in the current literature.

## 2. Case Presentation

A 35-year-old man with known atrial fibrillation, severe uncorrected congenital pectus excavatum (pectus index 4.8), and congenital left main coronary artery to right atrium fistula with a prior unsuccessful coil embolization presented with an accidental two-story fall. Preoperative risk assessment for trauma surgery prompted a coronary angiogram and right heart catheterization.

**Figure 1:**
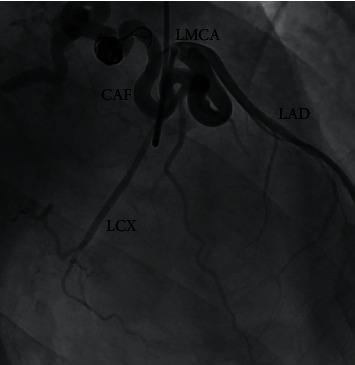
Coronary angiogram of the left main coronary artery (LMCA) showing filling of coronary arteriovenous fistula (CAF) originating near the LMCA bifurcation and connecting to the right atrium. LAD: left anterior descending artery; LCX: left circumflex artery.

Coronary angiography was performed via right radial artery approach showing a short left main coronary artery bifurcating into an intermediate size left anterior descending artery and left circumflex artery, along with filling of an expected coronary artery fistula seen as a large caliber tortuous vessel originating from left main coronary artery near bifurcation and draining into the right atrium. A previously deployed coil within the fistula was also seen but did not appear to offer any meaningful impedance to the fistula flow (Figure 1). Right heart catheterization was notable for normal right ventricular and normal pulmonary artery pressures. Mixed venous oxygen saturation measured from inferior and superior vena cava was 73%, and pulmonary artery oxygen saturation was 76%, resulting in a Qp/Qs ratio of 1.15, suggesting absence of any significant left-to-right shunt.

## 3. Discussion

Pectus excavatum, a posterior depression of the sternum and costal cartilages, is the most common congenital chest wall deformity, accounting for over 90% of such cases [1]. Although the exact etiology is not known, there appears to be a genetic contribution due to familial clustering of the disease [2]. In severe cases, right atrial and right ventricular compression from the deformity, as well as reduced lung volumes, result in decreased cardiac output and reduced exercise capacity [3]. Severity of pectus deformity is often measured using pectus index defined as the ratio of maximal transverse diameter to narrowest anterior posterior length of chest (distance between the vertebrae and sternum). A pectus index of greater than 3.0 is considered severe and often used to identify candidates for surgical correction in early years of life before cardiopulmonary complications develop [4]. Colombani showed that besides chest computed tomography and magnetic resonance imaging used for calculating pectus index and assessing the degree of cardiac compression, echocardiography provides additional information on diastolic chamber filling and cardiac function [5]. The author also proposed a criterion for selection of patients for surgical repair of pectus.

Coronary artery to right system fistulae of large size pose a risk of myocardial ischemia via steal phenomenon, as well as congestive heart failure and pulmonary hypertension by causing left-to-right shunt in most patients [6, 7]. Symptoms become apparent in a majority of young adults, frequently developing before the age of 20 [8]. While surgical or interventional treatment is usually reserved for symptoms of myocardial ischemia or congestive heart failure, American College of Cardiology and American Heart Association guidelines recommend that fistulae of large size should be treated regardless of symptoms or clinical manifestations (a class I indication) [9]. In addition, elective closure is also recommended for fistulae detected in early life and fistulae originating from proximal segments of coronary arteries, regardless of their size [7].

**Figure 2:**
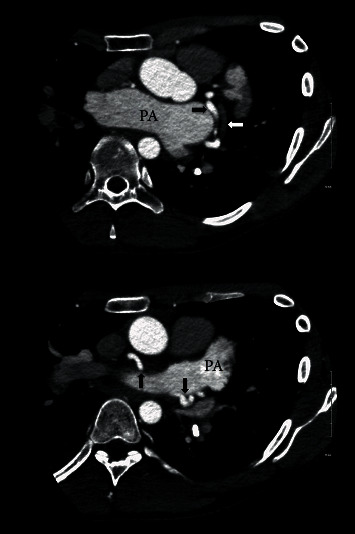
Thoracic computed tomography showing course of fistula (black arrows) originating in the left main coronary artery, initially running parallel to left circumflex artery (white arrow), posterior, and above the pulmonary artery (PA) before draining into the right atrium (right atrium not visualized in these sections).

**Figure 3:**
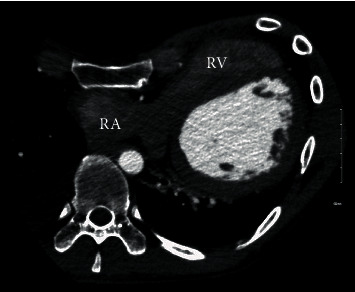
Computed tomogram showing pectus excavatum with severe compression and elongation of right atrium (RA) and right ventricle (RV).

In the case mentioned above, the patient was initially deemed a surgical candidate for pectus deformity as well as the coronary fistulae. Further workup including right heart catheterization and transthoracic echocardiogram revealed normal right-sided pressures and normal hemodynamics. A subsequent myocardial perfusion study excluded ischemia. Additionally, patient had maintained a normal functional and exercise capacity for over three decades of his life. This suggested that the coexistence of these two conditions likely resulted in a paradoxical balance, maintaining normal right-sided hemodynamics. Besides being an unusual case of a sizeable coronary fistula in a patient with severe pectus excavatum, the most remarkable aspect of this patient's pathophysiology is the likely neutralizing effect offered by his pectus deformity against development of right-sided overload and possibly vice versa (Figures 2 and 3). These findings led to a very carefully considered decision to allow both congenital deformities be left untreated in this case with no further intervention and conservative management was pursued. The patient remained asymptomatic on follow-up visit and continued to have normal exercise capacity.
